# Drug safety analyses in a rheumatoid arthritis registry: application of different approaches regarding timing of exposure and confounder measurement

**DOI:** 10.1186/s13075-017-1330-0

**Published:** 2017-06-13

**Authors:** Daniel H. Solomon, Nancy A. Shadick, Michael E. Weinblatt, Agnes Zak, Michelle Frits, Jessica M. Franklin

**Affiliations:** 1 Division of Rheumatology, Brigham and Women’s Hospital, 75 Francis Street, Boston, MA 02115 USA; 2 Division of Pharmacoepidemiology, Brigham and Women’s Hospital, Boston, MA 02120 USA

**Keywords:** Rheumatoid arthritis, Comparative effectiveness research, Disease-modifying antirheumatic drugs, TNF antagonist, Infection

## Abstract

**Background:**

Patient registry data serves an increasing role in drug safety and comparative effectiveness research, but registry databases often do not contain confounder information measured at the same time that treatments begin. This study evaluated a set of approaches for estimating confounder values at treatment initiation using actual data from a rheumatoid arthritis (RA) registry to examine the risk of infection associated with different biologic DMARDs (bDMARDs).

**Methods:**

We examined the risk of infection associated with starting a TNF inhibitor (TNFi) versus any of the other non-TNFi bDMARDs. Different confounder assessment approaches were tested. All approaches were tested in Cox proportional hazard regression models that used a propensity score (PS). The confounder of interest was the disease activity score (DAS28-CRP). The confounder assessment approaches utilized different temporal relationships between the DAS28-CRP measurement and the start of the treatments of interest.

**Results:**

We included 219 subjects with RA with 269 initiations of either a TNFi or a different bDMARD or both. Among this group, 305 infections were reported and confirmed through chart review. The hazard ratio (HR) for the risk of infection associated with use of a non-TNFi bDMARD ranged from 1.17 to 3.03 using 13 different approaches; only the approach with the highest HR produced results significantly different than one, but this approach included the fewest subjects and infections.

**Conclusions:**

The relative risk of infection for TNFi and other non-TNFi bDMARDs was similar using various approaches regarding which DAS28-CRP score should be used as the baseline measure in adjusted analyses.

**Electronic supplementary material:**

The online version of this article (doi:10.1186/s13075-017-1330-0) contains supplementary material, which is available to authorized users.

## Background

Clinical registries have grown in popularity over the last decade [1]. Their growth can be attributed to several factors. First, clinical registries allow for systematic collection of real-world practice data. Second, registry data provide evidence for comparative effectiveness and comparative safety evaluations. Third, registries facilitate patient-reported outcome data. The systematic collection of disease activity and patient-reported outcomes facilitates control for confounders in epidemiologic analyses. However, the confounders are not always measured at the ideal time.

For example, if one is interested in the relationship between a medication and an adverse outcome, it is ideal to have potential confounders measured at the time the medication was started. However, medications may be started between the time of registry visits and confounder collection. This raises the question of how to use confounder information collected at visits prior to or after the start of a given intervention.

We considered this methodologic question using a simulation study [2]. In the simulation, we examined 13 different approaches, including using confounder data from prior to the start of treatment, after the start of treatment, an average value, a weighted average value, and restricted cohorts based on proximity of the confounder measurement to the start of the intervention. In these simulations, we confirmed theoretical work showing that using the value of an important confounder measured after exposure start introduces bias for estimating the total effect of exposure on outcome if the confounder mediates part of the effect [3]. However, adjusting for the value measured from a visit prior to the start of exposure can also introduce bias, especially when measured long before exposure initiation. When selecting a time point for the confounder, analysts should carefully consider the timing of confounder measurements relative to exposure initiation and the rate of change in the confounder to choose the most relevant measure.

In the current set of analyses, we applied the simulation results using actual registry data to answer a relevant clinical question in the care of patients with rheumatoid arthritis (RA); namely, is use of non-tumor necrosis factor inhibitor (TNFi) biologic disease-modifying antirheumatic drugs (bDMARDs) associated with a higher risk of infection than use of a TNFi? Since patients with RA have a higher than expected risk of infection, [4] selecting treatments that minimize risk is a priority for patients and providers. The two strategies – use of a TNFi versus a non-TNFi bDMARD – are commonly considered strategies among patients with RA who have failed first-line disease-modifying antirheumatic drugs (DMARDs) [5]. While there are some data comparing these strategies in randomized controlled trials, those trials were relatively small and focused on efficacy not adverse events [6]. Since RA disease activity appears to be associated with infection risk, such analyses should include disease activity as a potential confounder. Thus, we have pursued study of this question in a registry of patients with RA.

## Methods

### Study design and cohort

The analyses conducted follow a simulation study examining different approaches to the measurement of confounders that change over time and may change in response to treatment. We tested a variety of scenarios and assumptions in a previous simulation study [2], and now apply the simulation results using actual data from the BRASS registry. BRASS is a single-center registry of patients with RA followed over the last 12 years [7]. Patients seen during routine clinical care who meet criteria for RA have been recruited and follow-up is conducted every 6 months. Follow-up includes standardized patient-reported outcome instruments, such as global arthritis activity, fatigue, functional status, and comorbid conditions [7–10].

Subjects selected for this analysis began either a TNFi (adalimumab, certolizumab pegol, etanercept, golimumab, infliximab) or non-TNFi bDMARD (e.g., abatacept, rituximab, tocilizumab) during BRASS follow-up. Four patients started tofacitinib, a newer synthetic DMARD, but not typically used first-line; these starts were not included. We required at least one BRASS visit prior to the start of the drug of interest as well as after.

### Data collection: outcomes and confounders

Since infections are an important potential adverse event in BRASS, we specifically ask about any infections since the last registry visit in the patient and physician questionnaires. Details such as type of infection, hospitalization, and treatments are collected. The dataset includes information on all patient- and physician-reported infections, from minor to severe. As part of this project, we performed a chart review on all such reported infections to assess which could be confirmed using the medical records from our health care system. This method would not necessarily ascertain information about infections that occur outside of our health care system. In the total cohort, we found medical record evidence to confirm (i.e., a provider note mentioning infection, microbiologic or radiographic information, or antibiotic prescriptions) an infection for 768 of the 1254 (61%) reported events. The primary outcome for this study was the confirmed infections and all reported infections were considered a secondary outcome.

The confounder of interest that we examined in these analyses using multiple assessment approaches is RA disease activity, as measured by the disease activity score based on 28 joints - C-reactive protein (DAS28-CRP) [11, 12]. This measure has been widely used in RA clinical research and includes four components: tender joint count, swollen joint count, patient global arthritis activity, and the C-reactive protein (CRP) level. When all components of the DAS28-CRP were not available, chart reviews were performed to extract missing data from the medical record. In 21 subjects, we were able to fill this information in from chart review. In one subject, last observation carried forward was used. For this confounder, we used 13 different approaches (see “Statistical analyses” below) for selecting the value to be used for adjustment.

For all other confounders, we used confounder information from the visit at the time the treatments of interest were started. However, in most instances, the treatments were started between registry visits. Thus, the covariates were assessed at the most proximal visit prior to the start of the treatment of interest.

### Statistical analyses

We examined the relationship between non-TNFi bDMARD use and infection, setting TNFi use as the reference exposure in a series of Cox proportional hazard regressions. To better balance baseline covariates, a propensity score was estimated using logistic regression [13]. The propensity score is the probability of one treatment or another, ranging from 0 to 1, based on a logistic regression that includes all of the potential confounders. We excluded subjects from the analysis with propensity scores that fell outside the distribution of the other exposure.

Separate Cox proportional hazard regression models were estimated for the various assessment approaches for the DAS28-CRP (see Table 1). The regression model included the exposure indicator, the propensity score as quintiles, as well as the DAS28-CRP. The adjusted hazard ratios were plotted for the primary outcome using an as-treated approach, in which patients are followed until the first censoring event. These included: first infection outcome; 30 days after the last dosage of a TNFi or a non-TNFi bDMARD; or loss to follow-up. A secondary analysis followed patients for a maximum of 6 months or one of the censoring events, whichever occurred first.

**Table 1:** Thirteen different approaches tested based on prior simulation results [2]

Approaches for DAS28-CRP adjustment	Description
1. No adjustment	No inclusion of any DAS28-CRP values.
2. Most recent prior measurement	Adjust for most recent DAS28-CRP value prior to start of treatment.
3. Most recent after measurement	Adjust for most recent DAS28-CRP value after start of treatment.
4. Arithmetic mean	Adjust for the arithmetic mean of the two nearest DAS28-CRP measurements.
5. Nearest measurement	Adjust for nearest DAS28-CRP measurement.
6. Weighted mean	Adjust for the weighted mean of the two nearest DAS28-CRP measurements, accounting for proximity.
7. Restricted to within 4 months	Only include subjects with DAS28-CRP within 4 months of start of treatment, prior or post.
8. Restricted to within 2 months	Only include subjects with DAS28-CRP within 2 months of start of treatment, prior or post.
9. Restricted to within 10 months prior	Only include subjects with DAS28-CRP within 10 months prior to start of treatment.
10. Restricted to within 8 months prior	Only include subjects with DAS28-CRP within 8 months prior to start of treatment.
11. Restricted to within 6 months prior	Only include subjects with DAS28-CRP within 6 months prior to start of treatment.
12. Restricted to within 4 months prior	Only include subjects with DAS28-CRP within 4 months prior to start of treatment.
13. Restricted to within 2 months prior	Only include subjects with DAS28-CRP within 2 months prior to start of treatment.

*DAS28-CRP* disease activity score based on 28 joints - C-reactive protein

Cox proportional hazard assumptions were tested by including time-dependent covariates in the Cox model. Interaction terms of each predictor and a function of survival time were added to the original Cox model and a proportionality test was performed. All time-dependent covariates were non-significant, as was the *p* value for the proportionality test, indicating that there is no evidence of violation of model assumptions. All analyses were conducted using SAS version 9.4 (SAS Institute Inc., Cary, NC, USA).

## Results

From the 1395 participants in BRASS, we identified 294 (21%) who were eligible for this study. Because of some baseline imbalance in covariates (see Additional file 1 for full cohort prior to trimming), a propensity score was calculated and the cohorts trimmed. Characteristics at the visit prior to the start of the medication of interest for the trimmed cohorts used in analyses are shown in Table 2. The two groups are well matched with respect to age, gender, serologic status, DAS28-CRP, concomitant non-biologic DMARDs, cigarette use, and diabetes. However, they differ in several baseline variables, including disease duration (other bDMARD 19 years vs TNFi 12 years), modified health assessment questionnaire (HAQ) (other bDMARD 0.6 vs TNFi 0.4), current glucocorticoid use (other bDMARD 57% vs TNFi 41%), and prior TNFi use (other bDMARD 92% vs TNFi 59%).

**Table 2:** Baseline characteristics in trimmed cohort

	Anti-TNF (n = 175)	Other bDMARD (n = 94)
	Mean (± SD) or median (IQR) or %	
Age, years	58 (±12)	60 (±11)
Female	86%	88%
Disease duration, years	12 (5, 26)	19 (10, 30)
Seropositive, RF or CCP	79%	85%
DAS28-CRP at T0	4.0 (2.9, 5.3)	4.4 (3.0, 5.4)
DAS28-CRP at T1	3.4 (2.4, 4.7)	3.9 (2.5, 5.2)
Modified HAQ score at T0	0.4 (0.1, 0.6)	0.6 (0.1, 1.0)
Modified HAQ score at T1	0.3 (0, 0.8)	0.5 (0.1, 0.8)
Corticosteroid use		
Current	41%	57%
In past 6 months	58%	79%
Ever	90%	96%
Cumulative glucocorticoid, mg	2464 (336, 12156)	6592 (1230, 17,546)
Prior TNF use	59%	92%
Concomitant non-biologic DMARD	69%	60%
Cigarette use, pack-years	0 (0, 13)	2.3 (0, 14)
Smoking status		
Never smoker	51%	47%
Past smoker	41%	51%
Current smoker	8%	2%
Diabetes	7%	9%

The only missing data were for cigarette use where nine subjects had missing values. T0 = visit immediately before start of treatment of interest; T1 = visit immediately after start of treatment of interest

*TNF* tumor necrosis factor, *bDMARD* biologic disease-modifying antirheumatic drug, *RF* rheumatoid factor, *CCP* cyclic citrullinated peptide, *DAS28-CRP* disease activity score based on 28 joints - C-reactive protein, HAQ health assessment questionnaire

The types of infections reported in BRASS by providers and patients are displayed in Table 3. Most of the infections were not severe and would not have required hospitalization. We found medical record evidence to confirm (i.e., a provider note mentioning infection, microbiologic or radiographic information, or antibiotic prescriptions) an infection for 305 of the 472 (65%) reported events in the trimmed cohort. The confirmed infections serve as the primary outcomes and the reported infections as the secondary outcomes for the regression analyses described below. For the primary outcome, the incidence rates per 100 person-years for infection for the TNFi group was 36.0 (95% CI 31.3 to 41.4) and 40.3 (95% CI 33.4 to 48.7) for non-TNFi bDMARDs.

**Table 3:** Infections reported in BRASS and confirmed through chart review

Infection type	Reported in BRASS^a^	Confirmed by chart review In total cohort	Confirmed by chart review In trimmed cohort^b^
	N (column %)		
Upper respiratory infection	274 (36%)	188 (25%)	84 (28%)
Sinus infection	207 (27%)	100 (13%)	48 (16%)
Genitourinary tract	163 (21%)	90 (12%)	34 (11%)
Bronchitis/pneumonia	154 (20%)	96 (12%)	46 (16%)
Skin, soft tissue	123 (16%)	173 (23%)	64 (21%)
Otitis media	33 (4%)	12 (2%)	4 (1%)
Bone or joint	27 (4%)	23 (3%)	4 (1%)
Blood stream	9 (1%)	10 (1%)	4 (1%)
Gastrointestinal	---	26 (3%)	4 (1%)
Oral/dental	---	15 (2%)	4 (1%)
Ophthamalogic	---	9 (1%)	2 (1%)
Lyme disease	---	7 (1%)	4 (1%)
Unknown source	264 (34%)	19(3%)	3 (1%)
Total	1254	768	305

^a^The infections reported in BRASS, by patients or rheumatology providers, were used in the sensitivity analysis reported in Fig. 2a. Columns may not add up to 100% because of rounding

^b^This column only includes infections occurring after the start of a new DMARD; these infections were used in the main analysis

We examined the distribution of DAS28-CRP measures recorded before and after the start of the medications of interest. The time between the visit immediately before the start of the medication of interest was similar in the two groups, 20–22 weeks (*p* = 0.37). Similarly, the time after the start of medication until the next study visit was similar in the two groups, 24–32 weeks (*p* = 0.39). In addition, we show the distribution of the actual DAS28-CRP scores before and after the start of the medications of interest (see Table 4 and Additional file 2). These show that DAS28-CRP did not change over time during the 12 months before the initiation of the treatment of interest, but there was a small decrease on average during the year after initiation. Therefore, measuring disease activity up to 12 months prior to treatment start may provide adequate confounding adjustment, while measurements after treatment start may deserve more scrutiny, as the observed decrease may be due to treatment.

**Table 4:** Distribution of DAS28-CRP scores used in analyses

Months BEFORE start of DMARD of interest	N	Mean DAS28-CRP (± SD)
0	46	4.1 ± 1.6
1	19	4.8 ± 1.5
2	18	4.7 ± 1.3
3	21	4.0 ± 1.6
4	28	3.6 ± 1.6
5	13	3.8 ± 1.8
6	11	3.8 ± 1.5
7	21	4.0 ± 1.9
8	21	4.1 ± 1.2
9	22	4.3 ± 1.3
10	15	4.2 ± 1.4
11	16	4.1 ± 1.4
12+	18	4.3 ± 1.9
Months AFTER start of DMARD of interest	N	Mean DAS28-CRP (± SD)
0	3	5.6 ± 0.8
1	21	4.2 ± 1.5
2	29	4.2 ± 1.5
3	25	3.8 ± 1.5
4	20	4.3 ± 1.3
5	16	3.6 ± 1.6
6	12	2.7 ± 1.0
7	14	3.4 ± 1.6
8	26	3.7 ± 1.3
9	25	3.8 ± 1.6
10	20	3.0 ± 1.2
11	19	4.0 ± 1.6
12+	39	3.1 ± 1.4

*DAS28-CRP* disease activity score based on 28 joints - C-reactive protein, *DMARD* disease-modifying antirheumatic drug

**Fig. 1:**
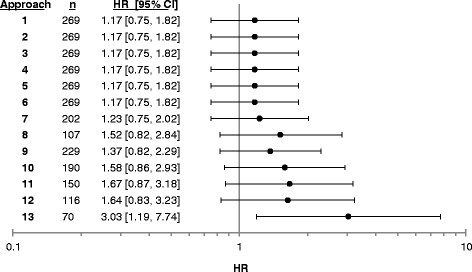
This graph displays the hazard ratios (*black circles*) and 95% confidence intervals (*lines through circles*) for each approach, using our primary outcome and analysis strategy

Using Cox proportional hazards regression, we estimated the hazard ratios across the 13 approaches for infection comparing TNFi (reference) to non-TNFi bDMARDs. Unadjusted risk was 1.17 (95% CI 0.75–1.82) (see Fig. 1). The approaches that included all subjects (scenarios 2–6) found very similar HRs. However, the HRs varied in approaches 7–13 when the analyses were restricted to smaller subsets of subjects with DAS28-CRP measured within a given proximity to the start of treatment. The most restrictive approach that only included patients with a DAS measurement 2 months prior to treatment start had just 70 observations and 36 infections, resulting in a strongly protective estimated effect of TNFi, but with poor precision.

**Fig. 2:**
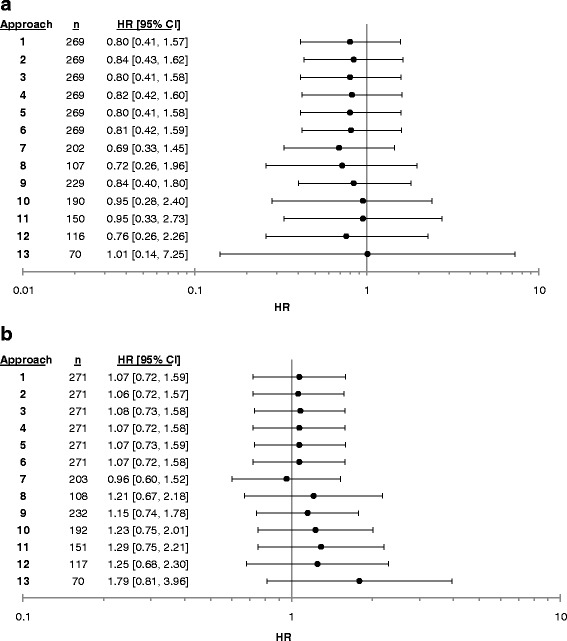
This graph displays the hazard ratios (*black circles*) and 95% confidence intervals (*lines through circles*) for each approach, using our secondary outcome (panel **a**) and secondary analysis strategy (panel **b**)

The HRs and patterns observed in the primary analyses remained stable in sensitivity analyses that included the secondary outcome of reported infection (see Fig. 2a) and the extended follow-up period (see Fig. 2b).

## Discussion

Registries are playing an increasing role in determining the post-marketing comparative safety of drugs. They have contributed important information to understanding the safety of various treatments for rheumatoid arthritis (RA). However, many methodologic questions regarding optimal analytic strategies have not been adequately answered for registries. Registries often include data on potential confounders measured at time points separate from when drugs are started. To assess the effect of different assumptions regarding how to estimate confounders in such circumstances, we focused on the comparative risk of infections among patients with RA starting different bDMARDs – any TNFi versus non-TNFi bDMARDs – using data from a single-center RA registry. We found that all approaches that estimated treatment effect using adjusted models in the full sample (approaches 2–6), regardless of the confounder assessment approach, gave very similar estimates. However, using restricted populations with disease activity measured in close temporal relationship before the start of drug (approaches 9–13) gave less precise estimates because of smaller sample sizes that indicated a stronger protective effect than other estimates. Similar results were also seen in the prior simulation study [2] and suggest that the risk of adverse effects of drugs differs across study populations in important ways, but based on subtle differences in the subpopulations of patients. While it may not be “incorrect” to estimate risks in subpopulations, the risks may differ substantially from the risk observed in the broader population.

The findings of this study have several methodologic implications. First, it is likely the case that the findings in this example apply to other registries and other clinical scenarios. We anticipate that whenever there is the possibility of a strong confounder, timing of its measurement with relation to the start of interventions is important and may influence estimates of association. Second, if one restricts analyses to subpopulations, even based on seemingly uninformative issues like when a confounder is measured, findings may not generalize to all subjects exposed to a given drug. This also may be due to the fact that the propensity score, estimated in the full sample, does not balance the confounder distributions as well in the subgroups identified in methods 7–13 (see Additional file 3: Table S2). Finally, as shown in the companion simulation study [2], it may be preferred to use confounder values from visits after the start of interventions if they are closer than any visit pre-intervention, especially if confounder values are changing rapidly prior to treatment initiation. This may seem to violate basic epidemiologic teaching that suggests that confounder measurement should always come before the start of an exposure [14].

Limitations of the current study include incomplete medical record data on all infections. However, analyses using the broader outcome of all reported infections gave us very similar results. As well, there were some missing data in the registry regarding disease activity. This was minimal. Patients may have differentially reported infections based on the time between starting a medication and the follow-up visit. Six months was the standard follow-up and this may have limited our ability to detect later effects of drug. It is also possible that disease activity was not a strong confounder in the clinical scenario under question. Prior data suggest disease activity impacts infection risk but the effect on treatment choice may not be as dramatic when choosing between different DMARDs. Strengths include the ability to test a prior set of simulation analyses in real data, the clinical relevance of the study question, the consistency of results across primary and secondary analyses, as well as the ability to confirm infections using medical records.

In addition to the methodologic implications of our findings, we must consider the potential clinical ramifications. The infection risk in this well-characterized cohort of RA patients starting new treatments did not differ between patients starting a TNFi and those starting other bDMARDs. These results are similar to a recently published large claims-based Medicare analysis that examined infection risk for each bDMARD separately [15]. This larger analysis found that infliximab and rituximab users had higher infection risk than abatacept, while other agents were similar in risk. The current study cohort included few infliximab and rituximab users. Our findings’ agreement with prior work is useful as a replication study using different methodology.

## Conclusions

In conclusion, we examined the risk of infection among patients with RA starting a TNFi versus a non-TNFi bDMARD. The analyses were conducted in the setting of a large single-center registry where the measurement of disease activity, a key potential confounder, often occurred at a different visit than the start of RA treatments. We tested various methods of estimating disease activity at treatment start and found that the risk of infection was similar for the two groups in nearly all approaches. This is in general agreement with prior estimates of risk [16]. Using all patients with the most proximal disease activity measurements, whether prior to or after the start of bDMARD, were most consistent, while analyses that restricted to subgroups with confounder data measured in very close proximity to the start of treatment appeared to give biased estimates with the least precision. The methods pursued in this study provide a template for how to evaluate the measurement of an important time-varying confounder in other registry analyses.

## Additional files

Additional file 1: Table S1. Baseline characteristics of subjects prior to trimming. Table describing baseline characteristics of subjects prior to trimming. (DOCX 13 kb)

Additional file 2: Figure S1. Change in DAS28-CRP between visits before and after start of treatment. Figure describing change in DAS28-CRP. (DOCX 463 kb)

Additional file 3: Table S2. Balance of selected potential confounders by exposure group for quintiles in methods 7–13. (DOCX 15 kb)

## Abbreviations

bDMARD: Biologic disease-modifying antirheumatic drug
CRP: C-reactive protein
DAS28: Disease activity score based on 28 joints
DMARD: Disease-modifying antirheumatic drug
RA: Rheumatoid arthritis
TNF: Tumor necrosis factor
