# Supplementary material for: Drug safety analyses in a rheumatoid arthritis registry: application of different approaches regarding timing of exposure and confounder measurement
Source: Arthritis Res Ther. 2017 Jun 13;19:130. doi: 10.1186/s13075-017-1330-0 (PMC5470201; doi:10.1186/s13075-017-1330-0)
Supplement: Supplementary file 1 — Baseline characteristics of subjects prior to trimming. Table describing baseline characteristics of subjects prior to trimming. (DOCX 13 kb) [file 13075_2017_1330_MOESM1_ESM.docx]

**Additional File 1: Table S1. Baseline characteristics of subjects prior to trimming**

| Covariates | Anti-TNF (n = 266) | Other bDMARDs (n = 99) |
| --- | --- | --- |
|  | Mean (± SD) or Median (IQR) | |
| Age | 56 (± 13) | 60 (± 11) |
| Female | 85% | 89% |
| Disease activity score at T0 | 4.2 (3.0, 5.4) | 4.3 (2.9, 5.3) |
| Disease activity score at T1 | 3.3 (2.2, 4.6) | 3.9 (2.5, 5.2) |
| Disease duration, years | 9 (3, 21) | 18 (10, 30) |
| Modified HAQ Score at T0 | 0.4 (0.1, 0.6) | 0.6 (0.1, 1.0) |
| Modified HAQ Score at T1 | 0.3 (0, 0.6) | 0.5 (0.1, 0.8) |
| Corticosteroid use |  |  |
| Current | 32% | 60% |
| In past 6 months | 50% | 80% |
| Ever | 86% | 96% |
| Cumulative steroid, milligrams | 1628 (224, 6480) | 6748 (1230, 17869) |
| Cigarette use, pack-years | 0 (0, 11) | 2.6 (0, 15) |
| Smoking status |  |  |
| Never | 53% | 45% |
| Past | 40% | 52% |
| Current | 7% | 3% |
| Seropositive, RF or CCP | 77% | 86% |
| Diabetes | 6% | 9% |
| Prior TNF Use | 47% | 92% |
| Concomitant DMARD | 73% | 58% |

Notes: The only missing data were for cigarette use where 10 subjects had missing values.
