# Supplementary figures and images for: Drug safety analyses in a rheumatoid arthritis registry: application of different approaches regarding timing of exposure and confounder measurement
Source: Arthritis Res Ther. 2017 Jun 13;19:130. doi: 10.1186/s13075-017-1330-0 (PMC5470201; doi:10.1186/s13075-017-1330-0)

**Additional File 2: Figure S1. Change in DAS28-CRP between visits before and after start of treatment.**


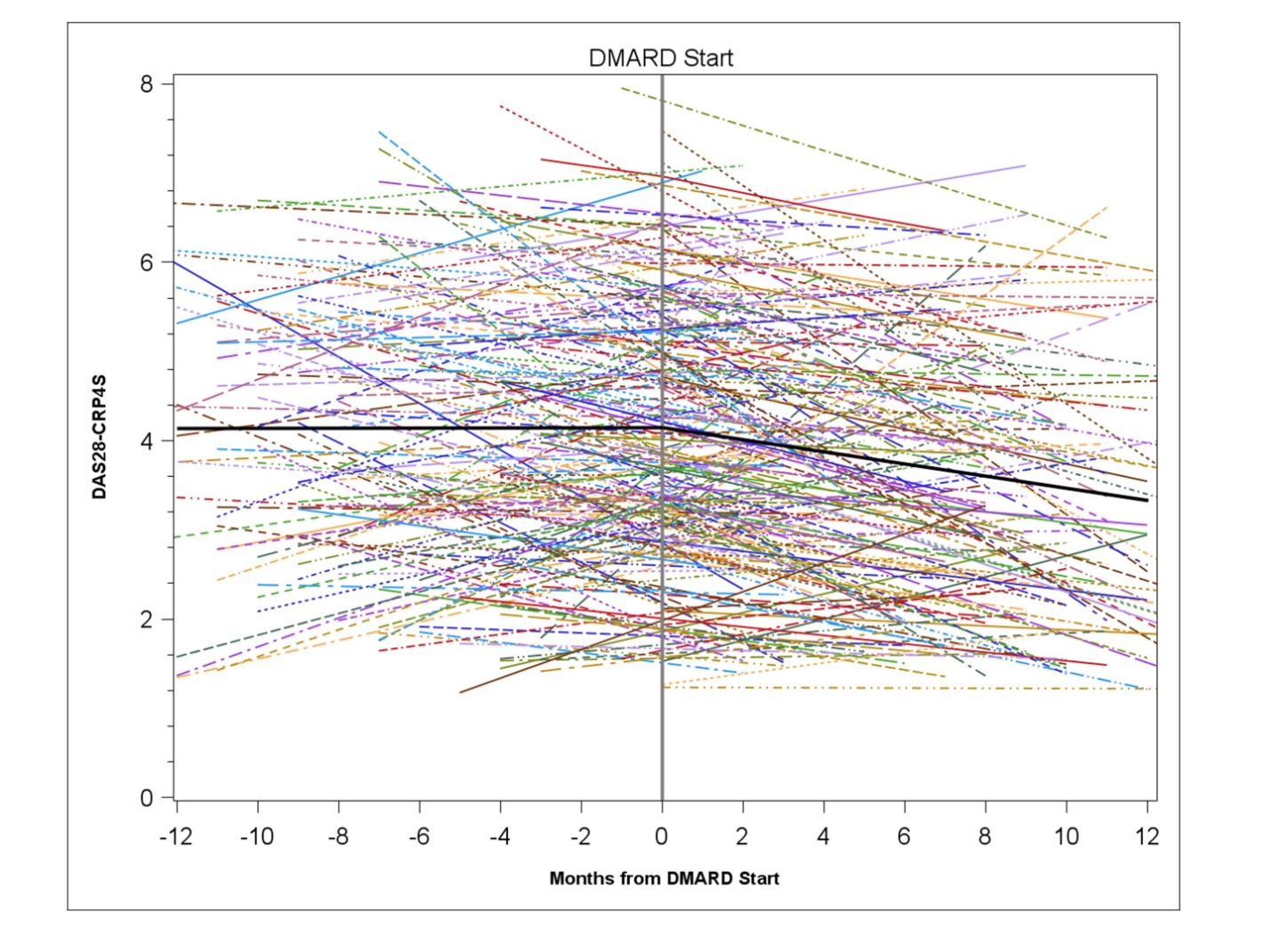

Supplement: Supplementary file 2 — Change in DAS28-CRP between visits before and after start of treatment. Figure describing change in DAS28-CRP. (DOCX 463 kb) [file 13075_2017_1330_MOESM2_ESM.docx]
