# Supplementary material for: Drug safety analyses in a rheumatoid arthritis registry: application of different approaches regarding timing of exposure and confounder measurement
Source: Arthritis Res Ther. 2017 Jun 13;19:130. doi: 10.1186/s13075-017-1330-0 (PMC5470201; doi:10.1186/s13075-017-1330-0)
Supplement: Supplementary file 3 — Balance of selected potential confounders by exposure group for quintiles in methods 7–13. (DOCX 15 kb) [file 13075_2017_1330_MOESM3_ESM.docx]

Additional file 3: Table S2: Balance of Selected Potential Confounders by Exposure Group for Quintiles in Methods 7-13

|  | Age, years [mean(sd)] | | Gender, % female | | Serostatus, % pos | | Diabetes, % yes | |
| --- | --- | --- | --- | --- | --- | --- | --- | --- |
| Method | TNFi | nonTNFi | TNFi | nonTNFi | TNFi | nonTNFi | TNFi | nonTNFi |
|  |  |  |  |  |  |  |  |  |
| **7** |  |  |  |  |  |  |  |  |
| Q1 | 62.5 (9.15) | 59.88 (13.69) | 83 | 85 | 92 | 88 | 8 | 8 |
| Q2 | 62.41 (13.42) | 60.04 (8.94) | 91 | 92 | 82 | 84 | 5 | 8 |
| Q3 | 57.59 (11.26) | 55.07 (12.68) | 79 | 93 | 72 | 87 | 10 | 7 |
| Q4 | 57.97 (10.26) | 68 (4.36) | 87 | 67 | 81 | 100 | 13 | 0 |
| Q5 | 56.57 (12.4) | 64 (13.44) | 80 | 75 | 80 | 75 | 6 | 25 |
| **8** |  |  |  |  |  |  |  |  |
| Q1 | 63.57 (9.52) | 58.23 (13.68) | 71 | 92 | 86 | 77 | 14 | 8 |
| Q2 | 64 (14.25) | 60.17 (9.92) | 86 | 100 | 86 | 92 | 7 | 8 |
| Q3 | 58.75 (8.95) | 55.5 (13.71) | 69 | 90 | 69 | 90 | 0 | 0 |
| Q4 | 59.33 (9.92) | 70 (.) | 92 | 100 | 75 | 100 | 25 | 0 |
| Q5 | 56.42 (14.47) | 67.33 (14.29) | 84 | 67 | 84 | 67 | 5 | 33 |
| **9** |  |  |  |  |  |  |  |  |
| Q1 | 59.25 (11.21) | 59.18 (12.58) | 92 | 88 | 100 | 88 | 8 | 9 |
| Q2 | 63.62 (13.29) | 59.73 (8.96) | 86 | 95 | 90 | 82 | 5 | 14 |
| Q3 | 55.85 (11.83) | 55.67 (11.74) | 82 | 100 | 70 | 83 | 6 | 8 |
| Q4 | 56.48 (12.48) | 64.8 (5.4) | 88 | 80 | 79 | 100 | 10 | 0 |
| Q5 | 56.86 (12.36) | 52.2 (10.16) | 84 | 80 | 73 | 60 | 7 | 20 |
| **10** |  |  |  |  |  |  |  |  |
| Q1 | 58.63 (12.69) | 60.52 (12.94) | 88 | 85 | 100 | 85 | 0 | 7 |
| Q2 | 64 (13.55) | 58.44 (9.38) | 89 | 94 | 89 | 81 | 6 | 13 |
| Q3 | 56 (11.52) | 59.22 (6.48) | 81 | 100 | 73 | 78 | 8 | 11 |
| Q4 | 57.44 (12.99) | 64.8 (5.4) | 86 | 80 | 78 | 100 | 11 | 0 |
| Q5 | 57.45 (12.5) | 52.2 (10.16) | 85 | 80 | 70 | 60 | 8 | 20 |
| **11** |  |  |  |  |  |  |  |  |
| Q1 | 58.63 (12.69) | 59.21 (14.09) | 88 | 89 | 100 | 79 | 0 | 5 |
| Q2 | 64.75 (13.54) | 57.46 (9.64) | 88 | 92 | 88 | 77 | 6 | 15 |
| Q3 | 55.3 (12.99) | 60.13 (6.29) | 80 | 100 | 80 | 75 | 10 | 13 |
| Q4 | 57.77 (12.55) | 64.8 (5.4) | 81 | 80 | 69 | 100 | 15 | 0 |
| Q5 | 57.68 (13.19) | 51.75 (11.67) | 81 | 75 | 71 | 50 | 6 | 25 |
| **12** |  |  |  |  |  |  |  |  |
| Q1 | 61.33 (9.71) | 60.36 (15.52) | 83 | 86 | 100 | 79 | 0 | 7 |
| Q2 | 64.73 (14.02) | 55.8 (9.95) | 87 | 90 | 87 | 80 | 7 | 20 |
| Q3 | 57.67 (12.1) | 60.13 (6.29) | 73 | 100 | 73 | 75 | 13 | 13 |
| Q4 | 60.53 (11.62) | 68 (4.36) | 76 | 67 | 65 | 100 | 24 | 0 |
| Q5 | 58.85 (11.7) | 59.5 (6.36) | 81 | 100 | 77 | 100 | 8 | 50 |
| **13** |  |  |  |  |  |  |  |  |
| Q1 | 59.33 (11.72) | 57.82 (14.86) | 67 | 91 | 100 | 73 | 0 | 9 |
| Q2 | 64.55 (15.7) | 54.33 (8.12) | 82 | 100 | 91 | 83 | 9 | 17 |
| Q3 | 58.22 (9.77) | 59.8 (7.98) | 67 | 100 | 67 | 80 | 0 | 0 |
| Q4 | 62.56 (8.13) | 70 (.) | 89 | 100 | 67 | 100 | 33 | 0 |
| Q5 | 59.29 (13.83) | 64 (.) | 86 | 100 | 79 | 100 | 7 | 0 |
